# A253 BEHAVIOUR CHANGE CONSIDERATIONS TO IMPROVE PHYSICAL ACTIVITY PARTICIPATION AMONG INDIVIDUALS WITH QUIESCENT AND MILDLY ACTIVE IBD – A QUALITATIVE STUDY

**DOI:** 10.1093/jcag/gwad061.253

**Published:** 2024-02-14

**Authors:** B Oketola, S Webber, H Singh, M Kredentser, K Reynolds, G Restall

**Affiliations:** Internal Medicine, University of Manitoba, Winnipeg, MB, Canada; Internal Medicine, University of Manitoba, Winnipeg, MB, Canada; Internal Medicine, University of Manitoba, Winnipeg, MB, Canada; Internal Medicine, University of Manitoba, Winnipeg, MB, Canada; Internal Medicine, University of Manitoba, Winnipeg, MB, Canada; Internal Medicine, University of Manitoba, Winnipeg, MB, Canada

## Abstract

NOT PUBLISHED AT AUTHOR’S REQUEST

Please acknowledge all funding agencies listed below

Funding Agencies

CAGPfizer

